# Special Issue “Neuromodulatory Effects of Serotonin”

**DOI:** 10.3390/ijms26051970

**Published:** 2025-02-25

**Authors:** Yasemin M. Akay

**Affiliations:** Biomedical Engineering Department, University of Houston, 3517 Cullen Blvd, Houston, TX 77204, USA; ymakay@uh.edu

Serotonin in the nervous system is synthesized from tryptophan by a small number of neurons within the central nervous system. Those neurons branch profusely and innervate all of the nervous system by releasing serotonin in different manners by exocytosis from different compartments of their complex structures and, by doing so, regulate a variety of functions, including many behaviors.

The type of location where serotonin is released defines the way the serotonin passes the information in each region. It is released in small amounts in the presynaptic terminals, producing very fast, local, and short-lasting effects on specific postsynaptic targets that participate in immediate functions. However, extra-synaptic sites of these neurons, including the soma and the axons, slowly release massive amounts of serotonin that produce less localized and more dynamic interactions with the target neurons, where serotonin can reach distant and diverse targets by diffusion, and produce slow and long-lasting neuromodulatory effects, such as those that characterize emotions and social behavior. By displaying different modes of neurotransmitter release, small numbers of serotonergic neurons can regulate a multiplicity of functions in the nervous system.

In this editorial, I will give a summary of seven papers which have the same topics as the contributing papers to this Special Issue, termed “Neuromodulatory Effects of Serotonin”, but written by different researchers and published in different journals other than *IJMS*.

Overview of the Literature:

Methylphenidate (Ritalin) is the most prescribed psychoactive drug in children for the treatment of attention deficit hyperactivity disorder (ADHD). According to the results in study [1], oral methylphenidate at doses within the therapeutic range significantly increases extracellular dopamine (DA) in human brain. The increase in DA, caused by the inhibition of dopamine transporters by methylphenidate, elevates the DA release. This would enhance task-specific signaling, improving attention and decreasing distractibility in subjects with ADHD treated by methylphenidate.

This study shows, for the first time, significant increases in extracellular DA after oral MP in humans. Subjects with ADHD, in whom increased brain levels of Dopamine Transporter (DAT) are likely to result in the rapid removal of DA from the extracellular space, may exhibit deficits in DA that are corrected by treatment with oral methylphenidate (MP).

Multifunctional Ca^2+^/calmodulin-dependent protein kinase (CaM kinase), which is transiently expressed in COS-7 cells, is physiologically inactive when assayed without Ca^2+^ in study [2]. According to the results in this study, overexpression of a constitutive construct (T286U) activated a commonly recognized CaM kinase pathway, and showed a biological response in xenopus oocytes. The authors did not know whether the endogenous Xenopus CaM kinase is normally involved in germinal vesicle movement, although it has been shown in other forms of motility because of its ability to phosphorylate myosin light chains. However, the authors’ aim was to determine whether the CaM kinase can be functionally expressed in a biological system, and this has been successfully accomplished. Expression of the mutant constructs, such as H282DR283GQ284E, which is 67% Ca^2+^ independent or T286DK300E, which has reduced calmodulin binding, could be helpful in exploring the neurotransmitter releases and synaptic plasticity where CaM kinase plays an important role.

Serotonin transporter (SERT) significantly affects the activity of serotonergic neurons in the central nervous system. The manipulation of SERT has long-term neurobiological and behavioral consequences, including developmental dysfunction, depression, anxiety, and auditory disorders. In study [3], the authors investigated the neuronal morphology and function of auditory cortex in SERT knockout (KO) mice. Results confirm that the number and density of dendritic spines of these neurons was significantly less than those of wild-type neurons. Also, the frequency tonotopic organization of primary auditory cortex was disrupted in SERT KO mice. These findings show that SERT plays a key role in development and functional maintenance of auditory cortical neurons. Therefore, the auditory function should be constantly checked when SERT is targeted in the treatment for psychiatric disorders.

Brain serotonin (5-HT) system dysfunction plays a role in depressive disorders, and acute depletion of 5-HT precursor tryptophan has frequently been used to model the influence of 5-HT deficiency on emotion regulation. In study [4], Tamoxifen (TAM)-induced Cre-/loxP-mediated inactivation of the tryptophan hydroxylase-2 gene (*Tph2*) was used to investigate the effects of provoked 5-HT deficiency in adult mice (*Tph2* icKO) previously subjected to maternal separation (MS). According to the results, inducible Trp hydroxylase knock-down (reducing 5-HT levels by 20%) impaired behavioral measures.

Conclusively, the authors’ findings developed an experimental model to study the behavioral and neurobiological consequences of 5-HT deficiency in adults with early-life stress experiences that potentially disrupt emotional stability and lead to the pathogenesis of depressive disorders.

In study [5], the researchers investigated the Catechol-O-Methyltransferase (COMT) Val^158^Met gene variation in relation to irrational beliefs, assuming this relationship depended on the level of emotional distress.

The authors focused on context-independent measures of irrational beliefs and emotional distress in the absence of a stressor. The results confirm the relationship between COMT Val^158^Met and irrational beliefs depends on the level of emotional distress.

These results were significant for overall irrational beliefs and their subtypes. Because of this study’s preliminary character, these findings should be checked by additional studies.

However, if replicable, the current findings might impact how COMT Val^158^ is related to treatment change in exposure-based, but not in cognitive-based, treatment methods.

In paper [6], the authors explored whether neonatal programming with sex hormones alters the response to morphine during adulthood in rats and makes them more sensitive to neurochemical, reward, and behavioral influences. According to the findings, there was a significant increase in morphine-induced dopamine release in the nucleus accumbens of rats that were exposed neonatally to estradiol or testosterone compared to control rats. The results suggested that early exposure to sex hormones could represent a vulnerability factor in the development of addictions to opioid drugs, such as morphine and heroin, in adulthood.

In conclusion, supra-physiological exposure to the sex hormones, or by persistent exposure to endocrine disruptors (environmental pollutants) with estrogenic activity in the early stages, produce long-term effects not only in reproductive tissues, but also in non-reproductive tissues, such as the brain. This study may also allow for exploration of the epigenetic mechanisms involved in DA neurons that may be responsible for the long-term effects observed in paper [6].

Studies in humans and animal models with spinal cord injuries (SCIs) have demonstrated that medications targeting serotonin receptors may decrease the vulnerability to central sleep-disordered breathing (SDB) [7]. The authors of this study investigated the effect of mirtazapine in decreasing the tendency to develop hypocapnic central sleep apnea (CSA) during sleep. Their findings suggest that the administration of mirtazapine can decrease the sensitivity to central apnea by reducing chemosensitivity and increasing carbon dioxide reserve.

To their knowledge, this research is novel, as it is the first study in humans evaluating the effect of mirtazapine on CO_2_ reserves and chemosensitivity in individuals with severe sleep-disordered breathing. This is also the first study to determine the potential therapeutic effects of mirtazapine on sleep parameters in individuals with a spinal cord injury.

Future studies using a greater sample size and a population with a more significant central apnea constituent should be conducted to acquire a better understanding of the clinical significance of mirtazapine treatment on SDB.

## References

[1] Volkow N.D., Wang G.-J., Fowler J.S., Logan J., Gerasimov M., Maynard L., Ding Y.-S., Gatley S.J., Gifford A., Franceschi D. (2001). Therapeutic Doses of Oral Methylphenidate Significantly Increase Extracellular Dopamine in the Human Brain. J. Neurosci..

[2] Waldmann R., Hanson P.I., Schulman H. (1990). Multifunctional Ca^2+^/Calmodulin-Dependent Protein Kinase Made Ca^2+^ Independent for Functional Studies. Biochemistry.

[3] Pan W., Pan J., Zhao Y., Zhang H., Tang J. (2021). Serotonin Transporter Defect Disturbs Structure and Function of the Auditory Cortex in Mice. Front. Neurosci..

[4] Aboagye B., Weber T., Merdian H.L., Bartsch D., Lesch K.P. (2021). Serotonin deficiency induced after brain maturation rescues consequences of early life adversity. Sci. Rep..

[5] Podina I., Popp R., Pop I., David D. (2015). Genetic Correlates of Maladaptive Beliefs: COMT VAL^158^MET and Irrational Cognitions Linked Depending on Distress. Behav. Ther..

[6] Velásquez V.B., Zamorano G.A., Martínez-Pinto J., Bonansco C., Jara P., Torres G.E., Renard G.M., Sotomayor-Zárate R. (2019). Programming of Dopaminergic Neurons by Early Exposure to Sex Hormones: Effects on Morphine-Induced Accumbens Dopamine Release, Reward, and Locomotor Behavior in Male and Female Rats. Front. Pharmacol..

[7] Prowting J., Maresh S., Vaughan S., Kruppe E., Alsabri B., Badr M.S., Sankar A. (2021). Mirtazapine reduces susceptibility to hypocapnic central sleep apnea in males with sleep-disordered breathing: A pilot study. J. Appl. Physiol..

